# Computed Tomography Imaging in Aortic Dissection

**DOI:** 10.5811/cpcem.2019.5.42531

**Published:** 2019-07-22

**Authors:** Amanda H. Westfall, John S. Garrett

**Affiliations:** Baylor University Medical Center, Department of Emergency Medicine, Dallas, Texas

## Abstract

Emergency physicians often rely on a “triple-rule-out” computed tomography (CT) where image acquisition is timed to obtain image quality equivalent to dedicated coronary CT angiography, pulmonary CT angiography, and thoracic aorta CT angiography. This case highlights the importance of obtaining CT angiography dedicated to the aorta in the setting of high clinical suspicion for aortic disease if initial CT pulmonary angiogram is negative for aortic disease.

## CASE PRESENTATION

**Image 1:**
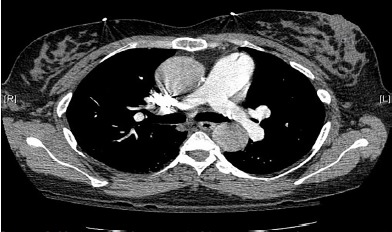
Unremarkable computed tomography pulmonary angiogram.

**Image 2:**
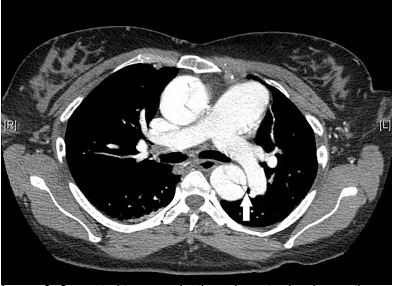
Computed tomography thoracic aorta showing aortic dissection (arrow).

A 39-year-old woman with twin gestation at 25 weeks presented to the emergency department with syncope. Her past medical history was significant for Marfan’s syndrome. Physical exam demonstrated confusion, hypotension, and a normal cardiopulmonary exam with equal pulses in all extremities. The patient denied chest pain. Differential diagnosis included aortic dissection and, given her pregnant state, pulmonary embolism. To minimize radiation exposure, a computed tomography (CT) pulmonary angiogram (PA) was obtained to evaluate for pulmonary embolism and aortic dissection (Image 1). Due to a negative CTPA and high suspicion for aortic disease, CT of the thoracic aorta was obtained (Image 2).

## DISCUSSION

### Type A Aortic Dissection

**Image 3:**
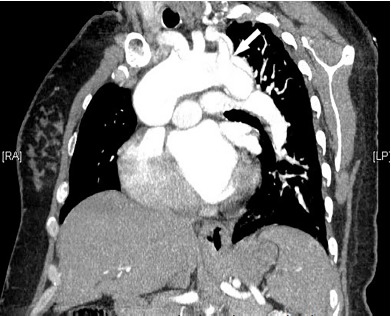
Computed tomography thoracic aorta showing aortic dissection involving left common carotid artery (arrow).

CT of the thoracic aorta revealed a dissection from the aortic root to the abdominal aorta and involving the left common carotid (Image 3). The patient was sent to the operating room for fenestration of the thoracoabdominal aorta and subsequent replacement of the ascending aorta and hemiarch. Her twins were delivered by emergent cesarean section at 28 weeks gestation. Mother was discharged to a rehabilitation facility and then home.

Thoracic CT imaging may evaluate for pulmonary embolism, aortic disease, and coronary artery disease. This can be accomplished through a “triple-rule-out” CT, where image acquisition is timed to obtain image quality equivalent to dedicated coronary, pulmonary and thoracic aorta CT angiography with high sensitivity and specificity.^1^ Emergency physicians should be aware of the significant limitations related to the contrast bolus timing, especially if a CTPA is used to screen for aortic pathology. If intravenous contrast is not within the aorta at the time of image acquisition, false negative results can occur, making aortic dissection invisible.^2^

**CPC-EM Capsule**

What do we already know about this clinical entity?

Type A Aortic dissection is a life-threatening disease with high morbidity and mortality. It requires early diagnosis, most often accomplished via computed tomography (CT) imaging.

What is the major impact of the image(s)?

These comparison images demonstrate the importance of obtaining CT angiography dedicated to the aorta, even if initial CT pulmonary angiogram is negative for aortic disease.

How might this improve emergency medicine practice?

These comparison images demonstrate the importance of obtaining CT angiography dedicated to the aorta, even if initial CT pulmonary angiogram is negative for aortic disease.
